# Suppurative complications following BCG vaccine in 2 Nepalese children

**DOI:** 10.1016/j.amsu.2022.103484

**Published:** 2022-03-14

**Authors:** Uttara Gautam, Ashish Lal Shrestha, Aakash Mishra

**Affiliations:** a Department of Pediatrics, Kathmandu Medical College Teaching Hospital, Kathmandu, Nepal; b Department of Pediatric and Neonatal Surgery, Kathmandu Medical College Teaching Hospital, Kathmandu, Nepal; c Kathmandu Medical College Teaching Hospital, Kathmandu, Nepal

**Keywords:** BCG complications, TB, BCG abscess, BCG adenitis

## Abstract

**Background:**

The Bacillus Calmette–Guérin (BCG) vaccine is one of the most common vaccines administered worldwide and awareness regarding its usual and adverse reactions is important. Local and systemic complications require accurate identification for timely therapy. We hereby report two patients with rare suppurative local complications of BCG.

**Case presentation:**

**Case 1:** A nine-month-old boy presented with swelling over the right deltoid for one month with low-grade fever and purulent discharge for two days. The active discharge occurred from the same site of previous BCG inoculation, the regression of which was achieved conservatively. **Case 2:** The second case was a 14-month-old boy who presented with a swelling over the lower part of the right axilla for one year, later diagnosed as a tuberculous lymph nodal abscess. Needle aspiration was done and anti-tubercular therapy was started based on positive Gene Xpert reports. Both the cases resolved completely without complications.

**Conclusion:**

Pharmacovigilance surveillance of BCG scar reactions and occurrence of suppurative complications should be known by clinicians for correct identification and management.

## Highlights

• BCG vaccine reduces the risk of both extra-pulmonary and pulmonary tuberculosis.
• Swelling and purulent discharge can occur months after BCG immunization.
• Tuberculous lymph nodal abscess is a rare complication of BCG.
• In lymph nodal abscess, pus drainage and ATT is done based on Gene Xpert reports.

## 1 Introduction

BCG vaccine contains a live attenuated strain of *Mycobacterium bovis* with an efficacy of 78% against disseminated tuberculosis (TB) and 64% against TB meningitis [1]. It reduces the risk of both pulmonary and extra-pulmonary TB by approximately 50% [2]. Normally given over the deltoid muscle as a single intradermal injection (0.05ml); it is usually prescribed for infants with a birth weight of over 2 kg.

An anticipated normal reaction is a red indurated area, which progresses onto a local lesion that may ulcerate 2–3 weeks after vaccination followed by encrustation in 3–4 weeks. At 6–10 weeks, the crust falls off, leaving a flat 3–7 mm scar. Axillary lymphadenopathy less than 1 cm is considered a usual response, just like non-suppurative lymphadenitis occurring within six months of vaccination with a resolution by nine months.

It is, however, crucial to distinguish this response from local complications like an abscess, suppurative lymphadenitis and keloid formation and systemic complications like disseminated TB that have all been recognized in the past [3]. This work has been arranged in line with the PROCESS 2020 guidelines [4].

## 2 Case Presentation

### 2.1 Case 1

A nine-month-old boy was brought with complaints of swelling over the right deltoid for one month associated with low-grade fever and purulent discharge for two days. He had been immunized with BCG vaccine over the same site at six weeks of age.

**Fig. 1:**
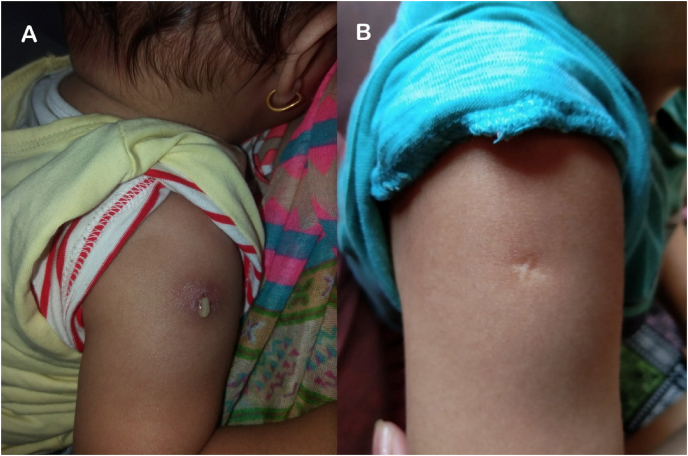
A. Active discharge from BCG inoculation site. B. Healed inoculation site with scar after two months.

On examination, he was not in obvious distress. Systemic examination was unremarkable. Local examination revealed a 2 × 2cm indurated area over the right deltoid with skin erythema and purulent discharge, the gram stain and culture of which were negative. Gene Xpert MTB/RIF assay and Acid-Fast Bacilli (AFB) smear and cultures were also negative. Regular wound dressings for two months led to a complete recovery, as shown in Fig. 1.

### 2.2 Case 2

**Fig. 2:**
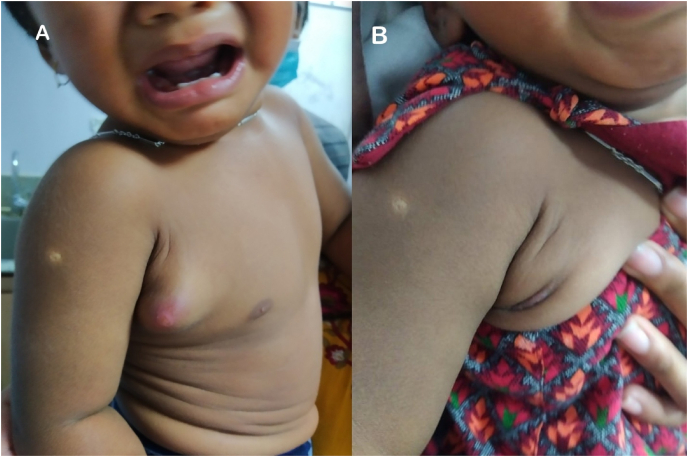
A. BCG scar over the right deltoid and accompanying lymph nodal abscess in the right axilla. B. Resolved axillary swelling.

A 14-month-old boy presented with a swelling over the lower part of the right axilla for one year. It was first noted at two months of age after BCG vaccination at the birth following which it had gradually increased in size with a rapid increase over the previous two months, as shown in Fig. 2A.

**Fig. 3:**
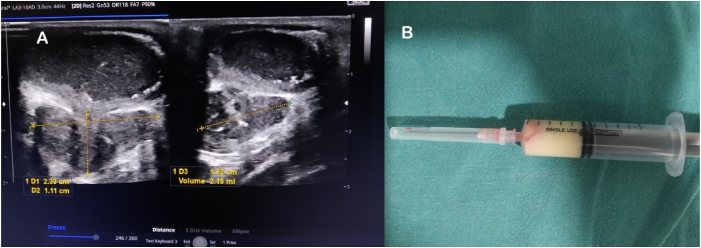
A. Ultrasound showing encapsulated lesion with iso to hyperechoic contents in the subcutaneous plane of the right axilla. B. Diagnostic aspiration showing thick pus.

Ultrasound of the right axilla revealed a well-defined, non-vascular, encapsulated lesion with iso to hyperechoic contents in the subcutaneous plane (Fig. 3A) the diagnostic aspiration of which showed thick pus (Fig. 3B). AFB stain and Gram stain of Pus were negative while Gene Xpert MTB RIF was reported to be positive. Also, total and differential blood count showed lymphocytosis.

A diagnosis of tuberculous lymph nodal abscess was made and anti-tubercular therapy was started as per national TB guidelines of Nepal. Isoniazid, Rifampicin, Pyrazinamide and Ethambutol were used for the first two months as intensive phase therapy, followed by Isoniazid and Rifampicin for the next 4 months as continuation phase treatment.

The boy was followed up regularly and noted to have a complete recovery (Fig. 2B).

## 3 Discussion

The World Health Organization (WHO) recommends BCG vaccination for all infants in countries with a high TB burden, wherein TB is a leading cause of death from a single infectious agent (ranking above HIV/AIDS) [5]. The National TB Prevalence Survey (TBPS) 2018-19 had shown around 117,000 people living in Nepal with TB (WHO 2019) [6].

In Nepal, the BCG vaccination campaign dates back to 1979 A.D. under the expanded program of immunization of WHO. With an ambitious project to end TB epidemic by 2050, it remains a cornerstone.

Local complications of BCG occur in approximately 1:1000 children and are present usually before six months of age [3,7]. This is probably related to immature immunity, causing an increased risk of developing local complications at younger ages. Local complications include 1. injection site reaction/abscess and 2. Non-suppurative lymphadenitis. The former can develop up to 30 days of injection with complete resolution within six months [8]. A conservative approach is usually adopted for this. The latter again considered a part of the normal course resolves spontaneously over a few weeks to months without sequelae [3,9]. Ipsilateral axillary nodes are the most common; supraclavicular, nuchal and cervical nodes have all been described in reports [9]. In a study from Japan, lymphadenopathy was detected in 79% of vaccinated children, the great majority resolving spontaneously and 0.02% proceeding to suppuration and discharge [8].

The management of suppurative BCG lymphadenitis, however, is not well defined with varied treatment approaches between conservative management, anti-tuberculous therapy (ATT), node aspiration, or combined ATT and aspiration [10].

Somehow, both our patients developed these complications after 6 months. The first patient was managed with regular wound dressings since AFB stain, Gram stain and culture were all negative. However, for the second patient, needle aspiration was done and anti-tubercular therapy was started based on positive Gene Xpert. Both the cases resolved completely without complications.

Considering its widespread use in developing nations, it seems important to be aware of normal response and be able to distinguish it from the local or systemic adverse reaction following BCG vaccination. This is helpful to avoid unnecessary alarms and guide the further course of management.

## Funding

None.

## Ethical approval

Not required.

## Consent for publication

Written informed consent was obtained from the patient's guardians for publication of this case report and accompanying images. A copy of the written consent is available for review by the Editor-in-Chief of this journal on request.

## Authors’ contribution

UG drafted the manuscript and involved in patient care. ALS was the treating physician, senior author and supervisor. AM revised the manuscript.

## Registration of research studies

Not Applicable

## Guarantor

Ashish Lal Shrestha is the guarantor of this article.

## Provenance and peer review

Not commissioned, externally peer reviewed.

## Declaration of competing interest

None to be declared.
